# Evaluation of surgical anti-adhesion products to reduce postsurgical intra-abdominal adhesion formation in a rat model

**DOI:** 10.1371/journal.pone.0172088

**Published:** 2017-02-16

**Authors:** Long-Xiang Lin, Fang Yuan, Hui-Hui Zhang, Ni-Na Liao, Jing-Wan Luo, Yu-Long Sun

**Affiliations:** Shenzhen Institutes of Advanced Technology, Chinese Academy of Sciences, Shenzhen, Guangdong, China; University of Louisville, UNITED STATES

## Abstract

**Background:**

Adhesions frequently occur after abdominal surgery. Many anti-adhesion products have been used in clinic. However, the evidences are short for surgeons to reasonably choose the suitable anti-adhesion produces in clinical practice. This study provided such evidence by comparing the efficiency of five products to prevent abdominal adhesion formation in a rat model.

**Methods:**

Fifty-six Sprague-Dawley rats were randomly divided into seven groups: sham-operation group, adhesion group, and five product groups (n = 8). The abdomens of rats were opened. The injuries were created on abdominal wall and cecum in the adhesion and product groups. The wounds on abdominal wall and cecum of rats in the adhesion group were not treated before the abdomens were closed. The wounds on abdominal wall and cecum of rats in the product groups were covered with anti-adhesion product: polylactic acid (PLA) film, Seprafilm^®^, medical polyethylene glycol berberine liquid (PEG), medical sodium hyaluronate gel (HA), or medical chitosan (Chitosan). Fourteen days after surgery, the adhesions were evaluated by incidence, severity, adhesion area on abdominal wall and adhesion breaking strength.

**Results:**

The application of PLA film and Seprafilm^®^ significantly reduced the incidence, severity, adhesion area and breaking strength of cecum-abdomen adhesion (P<0.05). HA, PEG and Chitosan failed to significantly reduce the cecum-abdomen adhesion (P>0.05). The statistical significances in the incidence and severity of abdomen-adipose adhesion between adhesion group and the product groups were not achieved. However, Seprafilm^®^ was more effective to reduce abdomen-adipose adhesion than PLA film. Furthermore, it was found that the products tested in this study did not effectively reduce cecum-adipose adhesion. The application of PEG could result in abdomen-small intestine adhesion.

**Conclusion:**

Based on the results of this study, the preference order of anti-adhesion products used to reduce postsurgical intra-abdominal adhesion formation is Seprafilm > PLA >> HA > Chitosan > PEG.

## Introduction

Postoperative adhesions, which could cause chronic pelvic pain, intestinal obstruction and infertility, often occur after abdominal surgery [1, 2]. A number of products, in the form of film or fluid, are widely used to prevent postoperative adhesion formation [3]. These products normally serve as barriers to separate the contact of the damaged tissue surfaces. The products commonly used in clinic are polylactic acid film, Seprafilm^®^, medical sodium hyaluronate, medical chitosan and so on. It was found that they were able to reduce adhesion formation in many animal models and some clinical practices [4–8]. However, few studies simultaneously compared the anti-adhesive efficiency of a broad list of the commercial products with the same animal model or clinical practice[9]. Therefore, there are few evidences for surgeons to choose the suitable products in their clinical practice. This study was to investigate the efficacy of five anti-adhesive products on preventing postoperative adhesion formation in a rat model. The purpose was to provide the guidance for surgeons to use anti-adhesion produces in their clinical practice.

## Materials and methods

### Materials

All anti-adhesive products used in this study were: Polylactic acid (PLA) film (Nocling^TM^ resorbable medical film, Shanghai Divine Medical Technology Co., Ltd, China), Seprafilm^®^ adhesion barrier (Genzyme Biosurgery, Framingham, MA, USA), Medical polyethylene glycol berberine liquid (PEG) (Nianlianping^®^, Heilongjiang Liaoyuan Science and Technology Co., Ltd, China), Medical Sodium Hyaluronate Gel (HA) (Shanghai Jianhua Fine Biological Products Co., Ltd, China), Medical Chitosan (Chitosan) (Chitogel^®^, Shanghai Qisheng Biological Preparation Co., Ltd, China).

### Animals

Fifty-six adult female Sprague-Dawley rats (230 to 280 g) used in this study were purchased from Guangdong Province Laboratory Animal Center (Guangzhou, China). The rats were treated in accordance with protocols approved by the Institutional Animal Care and Usage Committee at Shenzhen Institutes of Advanced Technology (SIAT) (File Number: SIAT-TRB-140613-YGS-SYL-A0076). Animals were housed for one week to acclimatize to the local environment before the surgery.

### Surgical procedures

Animals were randomly divided into seven groups: sham-operation, adhesion, PLA, Seprafilm^®^, PEG, HA and Chitosan groups (n = 8). The surgical procedure was performed as described previously [10]. All animals were anesthetized with pentobarbital sodium (40 mg/kg) by intraperitoneal injection. After abdominal hair was shaved, the ventral skin was sterilized with 5% iodophor. Under aseptic conditions, a 6 cm incision was made at the middle of abdominal skin longitudinally. Then a 5 cm incision along the median line of abdominal wall was made. The ceca of rats in sham-operation group were elevated out the abdomens, covered with wet gauze for about 8 min, and moved back without creating any extra injuries. The abdominal wall incision was closed with a running 4–0 polypropylene suture, and the skin was closed with 4–0 silk suture. In other groups, the homogeneously petechial hemorrhage with area of approximately 1 cm × 2 cm on surface of the cecum was abraded with a toothbrush by 100 strokes then covered with wet gauze. A 1 cm × 2 cm segment of parietal peritoneum, which was 1 cm lateral to the midline incision, was sharply dissected with a scalpel blade from the superficial layer of underlying muscle. The abdominal cavities of rats in adhesion group were closed without any treatment on the wounds. The wounds of the rats in the product groups were covered with anti-adhesion products: polylactic acid film (2 cm × 3 cm) which was fixed with sutures at its four corners for about 2 min, Seprafilm^®^ (2 cm × 3 cm), medical polyethylene glycol berberine liquid (0.6 ml), medical sodium hyaluronate gel (0.6 ml), or medical chitosan (0.6 ml). Then ceca were put back into the abdominal cavity. The two defects on the cecum and abdominal wall were placed in contact. Finally, the abdominal wall incision was closed with a running 4–0 polypropylene suture, and the skin was closed with 4–0 silk suture. A single person performed all surgical procedures with a same assistant. Animals were allowed to recover at cages for 2 weeks.

### Assessment of adhesions

At day 14 after surgery, the animals were euthanized with excessive sodium pentobarbital (120 mg/kg). The abdominal cavity was opened via a U-shaped incision. The severity and area of adhesion between the cecum and abdominal wall was evaluated according to a widely used scoring system (Table 1) [11]. The same scoring system was used to evaluate abdomen-adipose adhesion, cecum-adipose adhesion and abdomen-small intestine adhesion. The assessment was performed by the four investigators (FY, LXL, HHZ and YLS) blinded to the surgical procedures.

**Table 1:** Adhesion Severity and Adhesion Area Scoring Scheme.

Degree	Description	
	Adhesion Severity	Adhesion Area
0	No adhesions	No adhesions
1	Thin filmy adhesion	≤25% of initial injured area
2	More than one thin adhesion	25–50% of initial injured area
3	Thick adhesion with focal point	50–70% of initial injured area
4	Thick adhesion with planar attachment	75–100% of initial injured area

### Mechanical evaluation of cecum-abdomen adhesion

The separation of cecum-abdomen adhesion was carried out by a customized mechanical testing device [12]. The cecum-abdomen complexes were fixed on the device in the horizontal orientation. The cecum was clamped with a fixed clamp, while the abdominal wall muscle was pulled by an actuator at a speed of 1.9 mm/s. The adhesion breaking strength was measured during the separation of cecum and abdomen wall with a load cell. The samples were kept moisture during the mechanical test.

### Statistical analysis

Data presented as mean ± SD were analyzed with SPSS analysis software (version 19.0, SPSS Inc., Chicago, IL) in this study. Fisher exact test was used to compare the incidence of adhesions in the sham or adhesion group with other groups. Kruskal-Wallis rank test was used to analyze the adhesion severity and adhesion area among the different experiment groups. One-way ANOVA, using Tukey΄s honestly significant difference test as a post hoc test, was used to compare the differences of adhesion breaking strength among the experiment groups. P < 0.05 was regarded as statistical significance.

## Results

Adhesions were not found at the initial laparotomy. On the 14th day after surgery, the major adhesions identified in the study were cecum-abdomen adhesion, abdomen-adipose adhesion, cecum-adipose adhesion and abdomen-small intestine adhesion. The residues of products were not observed in the abdomen of rats except PLA. PLA film was enclosed with fibrous tissue and attached on the abdominal wall of 6 rats in PLA group.

The cecum-abdomen adhesion was the most severe adhesion of the adhesions identified in the adhesion group (Table 2). With the success of the model, the cecum-abdomen adhesion was not found in the sham group. All products could decrease the incidence and severity of cecum-abdomen adhesion more or less. Only 1 rat had cecum-abdomen adhesion in PLA and Seprafilm^®^ groups. The adhesion score of the rat in PLA group was a little higher than that of the rat in Seprafilm^®^ group. The cecum-abdomen adhesion was found in 6 rats in PEG group, and all were grade 4. Six rats in the HA group had cecum-abdomen adhesion; their adhesion severity was smaller than that in PEG group. The rats in Chitosan group had the highest incidence of cecum-abdomen adhesion among the product groups. Only 1 rat was free of adhesion. The statistical analysis of the incidence and severity of cecum-abdomen adhesion showed that PLA and Seprafilm^®^ significantly reduced incidence and severity of cecum-abdomen adhesion comparing to the adhesion group (P<0.05). PEG, HA and Chitosan could not effectively reduce cecum-abdomen adhesion (P>0.05).

**Table 2:** The severity and incidence of cecum-abdomen adhesion of rats in the experiment groups.

Rat	Group						
	Sham^△^	Adhesion*	PLA^△^	Seprafilm^△^	PEG*	HA	Chitosan*
1	0±0	4±0	0±0	0±0	0±0	0±0	4±0
2	0±0	4±0	0±0	0±0	4±0	3.5±0.58	3±0
3	0±0	4±0	0±0	0±0	4±0	4±0	0±0
4	0±0	4±0	0±0	0±0	4±0	2.5±0.58	4±0
5	0±0	4±0	0±0	0±0	0±0	4±0	4±0
6	0±0	4±0	0±0	0±0	4±0	0±0	2.5±1
7	0±0	4±0	3.25±0.5	2±1.15	4±0	4±0	4±0
8	0±0	4±0	0±0	0±0	4±0	3±0	4±0
Adhesion incidence (%)	0^△^	100*	12.5^△^	12.5^△^	75*	75*	87.5*

*P<0.05 (vs. Sham)

^△^P<0.05 (vs. Adhesion).

The cecum-abdomen adhesion was also evaluated with adhesion area on the abdominal wall (Table 3). No cecum-abdomen adhesion was formed in all rats in the sham group. Therefore their adhesion area scores were assigned as 0. All rats in the adhesion group had the adhesion with the most severe grade, grade 4. Only 1 rat in PLA group or Seprafilm^®^ group had the adhesion with grade 1. In the PEG group, two rats were free of the adhesion, two rats had the adhesion with grade 3 and other 4 rats had the adhesion with grade 4. The rats in HA group had the cecum-abdomen adhesion with less adhesion area on the abdominal walls than that in PEG group. The rats in Chitosan group had the similar adhesion area as that in PEG group. The statistical analysis obtained the same conclusion as the investigation of adhesion incidence and severity of cecum-abdomen adhesion. Only PLA and Seprafilm^®^ could significantly reduce the adhesion area comparing to the adhesion group.

**Table 3:** Adhesion area of rats in the experiment groups.

Rat	Group						
	Sham^△^	Adhesion*	PLA^△^	Seprafilm^△^	PEG	HA	Chitosan*
1	0±0	4±0	0±0	0±0	0±0	0±0	3.75±0.5
2	0±0	4±0	0±0	0±0	3±0	1.75±0.5	1±0
3	0±0	4±0	0±0	0±0	4±0	3±0	0±0
4	0±0	4±0	0±0	0±0	4±0	1±0	4±0
5	0±0	4±0	0±0	0±0	0±0	4±0	4±0
6	0±0	4±0	0±0	0±0	4±0	0±0	1±0
7	0±0	4±0	1±0	1±0	4±0	4±0	4±0
8	0±0	4±0	0±0	0±0	3±0	1±0	4±0

*P<0.05 (vs. Sham)

^△^P<0.05 (vs. Adhesion).

**Fig 1:**
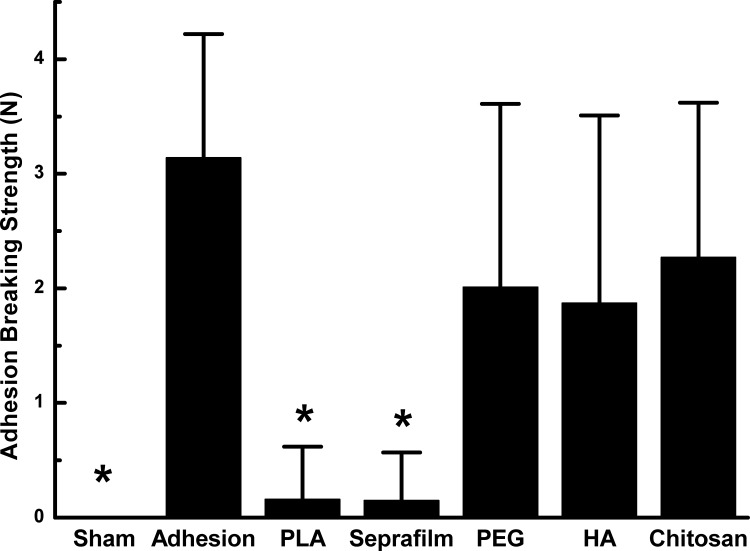
Adhesion breaking strength of cecum-abdomen adhesion in the different experimental groups. *P<0.05 vs adhesion group.

The cecum-abdomen adhesion formation was further evaluated with adhesion breaking strength (Fig 1). The adhesion breaking strength of the sham group was assigned 0 N as no adhesion occurred. The adhesion breaking strength of the adhesion group was 3.14±1.08 N, while the adhesion breaking strengths of the PLA and Seprafilm^®^ group were as small as 0.16±0.46 N and 0.15±0.42 N, respectively, which were significantly smaller than that of the adhesion group. The adhesion breaking strength of PEG (2.48±1.85 N), HA (1.87±1.64 N) or Chitosan (2.45±1.47 N) group was not significantly different from that of the adhesion group (P>0.05). The results also confirmed that application of PLA film and Seprafilm^®^ was superior to reduce the cecum-abdomen adhesion formation comparing to PEG, HA and Chitosan.

Other adhesions were evaluated in addition to the cecum-abdomen adhesion. The abdomen-adipose adhesion was not found in the rats in the sham group (Table 4). Five rats in the adhesion group had abdomen-adipose adhesion with the adhesion score 1 to 2. PLA could lead to more severe abdomen-adipose adhesion with a higher incidence comparing to the adhesion group. Seprafilm^®^ group had much less incidence of abdomen-adipose adhesion comparing to the adhesion group and other product groups. The statistical significance of the incidence and severity of abdomen-adipose adhesion was only achieved between PLA group and Seprafilm^®^ group in the study. The injuries of abdominal wall and cecum also could result in cecum-adipose adhesion (Table 5). It was found that all products tested in this study could not effectively reduce cecum-adipose adhesion. PLA led to more severe cecum-adipose adhesion with a higher incidence comparing to the adhesion group. Besides the adhesions listed above, the abdomen-small intestine adhesion was found in 4 rats in the study. All 4 rats were in the PEG group and the severity scores were 2 to 3.

**Table 4:** The severity and incidence of abdomen-adipose adhesion of rats in the experiment groups.

Rat	Group						
	Sham	Adhesion*	PLA	Seprafilm	PEG	HA	Chitosan
1	0±0	2±0	3.5±1	0±0	0±0	0±0	1.75±0.5
2	0±0	1.5±0.58	1.75±0.5	0±0	1.75±0.5	2±0	0±0
3	0±0	1±0	3±0.82	0±0	2±0	0±0	0±0
4	0±0	0±0	1.25±0.5	0±0	1±0	2±0.82	0±0
5	0±0	1.5±0.58	2±0	0±0	0±0	2±0	1.25±0.5
6	0±0	0±0	2.25±0.5	0±0	1±0	0±0	1.25±0.5
7	0±0	1.25±0.5	1.25±0.5	2.25±0.5	1.25±0.5	0±0	0±0
8	0±0	0±0	0±0	0±0	1.75±0.5	1±0	0±0
Adhesion incidence (%)	0^△^	62.5*	87.5*	12.5	75*	50	37.5

*P<0.05 (vs. Sham)

^△^P<0.05 (vs. Adhesion).

**Table 5:** The severity and incidence of cecum-adipose adhesion of rats in the experiment groups.

Rat	Group						
	Sham	Adhesion	PLA	Seprafilm	PEG	HA	Chitosan
1	0±0	2±0	0±0	1.75±0.5	2±0	2±0	2±0
2	0±0	2±0	1±0	0±0	2±0	2±0.82	2±0
3	0±0	0±0	0.5±1	1±0	2±0	0±0	2±0
4	0±0	0±0	2.25±0.5	1±0	0±0	2±0.82	2±0
5	0±0	2±0	3±0.82	0±0	2.5±1	1.75±0.5	0±0
6	0±0	1.25±0.5	1.75±0.5	0±0	0±0	0±0	0±0
7	0±0	1.25±0.5	2±0	2±0	0±0	0±0	0±0
8	0±0	0±0	2±0	2±0	2±0	1±0	0±0
Adhesion incidence (%)	0^△^	62.5*	87.5*	62.5*	62.5*	62.5*	50

*P<0.05 (vs. Sham)

^△^P<0.05 (vs. Adhesion).

## Discussion

The postsurgical abdominal adhesions remain a significant cause of morbidity for a large number of patients [13, 14]. Many products are applied in clinic to reduce the abdominal adhesion formation [3]. Seprafilm^®^ has been proved to be safe and effective in reducing postsurgical adhesions in a variety of animal models and clinical studies [4]. It was reported that the application of polylactic acid film significantly reduced intra-abdominal adhesions in clinic [5]. Hyaluronic acid (HA) is a natural linear polysaccharide. The cross-linked hyaluronan was found to have an efficacious anti-adhesive action following laparoscopic myomectomy in patients [6]. Chitosan is a highly charged polysaccharide with similar structure of hyaluronic acid. It was reported that medical chitosan could effectively reduce intra-abdominal adhesions after obstetric and gynecological surgeries [7]. PEG is a polyether compound prepared by polymerization of ethylene oxide. Some clinical studies provided the evidence of the efficacy of PEG for the reduction of adhesions following myomectomy [8, 15]. Although the effectiveness of the products to reduce abdominal adhesions was supported by a number of clinical studies, the controversial results were often reported [3]. In addition, it is unclear which products are superior to prevent postsurgical abdominal adhesion formation.

In this study, the efficiency of five commercial products to reduce postsurgical intra-abdominal adhesion formation was compared in a rat model. Our study revealed that PLA film and Seprafilm^®^ had the similar ability to effectively reduce the cecum-abdomen adhesion. This result is consistent with the observation in the previous study [9]. HA, chitosan and PEG only slightly reduced the cecum-abdomen adhesion. This study demonstrated that the film products (PLA film and Seprafilm^®^) had much better anti-adhesive performance comparing to the fluid products (PEG, HA and Chitosan). The higher adhesion incidence and severity of the fluid products could result from the nature of fluid. First, the fluid products can be diluted with blood which is commonly associated with the injuries. Second, the fluid products normally flow to other locations in the abdominal cavity instead of staying on the wounds during the procedure of surgery and the routine activities after the surgery. Finally, the fluid products do not have the sufficient mechanical strength as a physical barrier to separate the injured tissues.

The higher efficiency of film products to reduce cecum-abdomen adhesion could associate with that the film products have the better ability to separate the injured tissues. It was found that Seprafilm^®^ was completely degraded and the PLA film dislocated at 14th day after surgery. It indicates that the high efficiency of these two products to reduce the adhesion results from their ability to effectively separate the injured tissues at the early stage of healing. This finding agrees to the fact that the early period after surgeries is vital for the peritoneal adhesion formation [16]. Therefore, the effective separation of the injured tissues in the early period of healing is crucial to reduce cecum-abdomen adhesion.

PLA film resulted in more abdomen-adipose adhesion than Seprafilm^®^ in this study. It was reported that a prolonged surgery could cause peritoneal alterations and postoperative adhesion formation [17, 18]. A two-minute prolonged surgery was required for PLA film to be fixed with suture comparing to the surgical procedures of other groups in the study. The more abdomen-adipose adhesion in the PLA group than Seprafilm^®^ group could be attributed to the prolonged surgery. However, these two products had the same efficacy to reduce cecum-abdomen adhesion. Therefore, the slightly longer surgical procedure in the PLA group may have a minor effect on the adhesion formation. The difference of anti-adhesion abilities of these two products could result from the difference of their hemostatic capabilities. It was found that Seprafilm^®^ but not PLA film achieved hemostasis as soon as it was applied on the wounds. The less efficiency of PEG, HA and chitosan to reduce postsurgical intra-abdominal adhesion formation could also result from the low hemostatic capabilities of these fluid products.

Seprafilm^®^ and PLA film could effectively reduce the cecum-abdomen adhesion. However, they have a number of limitations. First, they could not effectively reduce all types of adhesion. It was found they had little effect to reduce cecum-adipose adhesion in this study. Second, it is difficult or impossible to use them in laparoscopic surgery and to apply to the tissues with the complex geometries. Third, PLA film has to be fixed by suture in the surgery. It takes more time in the surgeries. Fourth, PLA film was degraded slowly. The residue of PLA could lead more adhesions. Finally, it is difficult to handle Seprafilm^®^ in the surgical procedure. Once the Seprafilm^®^ attaches to the wet glove, devices or tissues, it becomes too fragile to be relocated. Therefore, it is still crucial to develop the new products to effectively reduce postsurgical adhesions in a broad range and to be easily used in clinic.

## Conclusion

Anti-adhesion products have been wildly used in clinic. Based on the results of this study, the preference order of the anti-adhesion products used to reduce postsurgical intra-abdominal adhesion formation is Seprafilm > PLA >> HA > Chitosan > PEG. All these products are still far from perfect for clinical use, it is necessary to develop the new anti-adhesion products.
